# A new Schiff base coordinated copper(II) compound induces apoptosis and inhibits tumor growth in gastric cancer

**DOI:** 10.1186/s12935-019-0801-6

**Published:** 2019-04-03

**Authors:** Yan Xia, Xingkai Liu, Luping Zhang, Jinzhu Zhang, Chaoying Li, Nan Zhang, Hong Xu, Yan Li

**Affiliations:** 1 Department of Gastroenterology, The First Hospital of Jilin University, No. 71. Xinmin Street, Changchun, 130021 Jilin China; 2 Department of Hepatobiliary and Pancreas Surgery, The First Hospital, Jilin University, Changchun, Jilin China; 3 College of Basic Medical Science, Jilin University, Changchun, 130021 China; 4 Division of Surgical Oncology, Department of Surgery, University of Louisville, Louisville, KY 40202 USA

**Keywords:** Schiff base copper coordination compound, NF-κB, Autophagy, ROS

## Abstract

**Background:**

Gastric cancer, as a multifactorial disorders, shows cytological and architectural heterogeneity compared to other gastrointestinal cancers, making it therapeutically challenging. Cisplatin is generally used in clinic for gastric cancer treatment but with toxic side effects and develops resistance. Anti-tumor properties of copper and its coordinated compounds have been explored intensively in recent years.

**Methods:**

In this study, we synthesized a novel Schiff base copper coordinated compound (SBCCC) and examined its antitumor effects in two gastric cancer cell lines SGC-7901 and BGC-823 as well as a mouse model of gastric cancer.

**Results:**

The results show that SBCCC can significantly inhibit the proliferation of gastric cancer cells in a dose- and time-dependent manner. The IC50 of SBCCC in SGC-7901 and BGC-823 cells is 1 μM, which is much less than cisplatin’s IC50. SBCCC induces apoptosis and causes cell cycle arrest at the G1 phase. SBCCC induces apoptosis via multiple pathways including inhibition of NF-κB, ROS production and autophagy.

**Conclusions:**

The synthesized SBCCC induced cancer cell death via inhibition of NF-κB, ROS production and autophagy. The multiple cell-killing mechanisms were important to overcome therapeutic failure because of multidrug-resistance of cancer cells. SBCCC, with a lower IC50 compared to cisplatin, could render it the potential to overcome the side-effect for clinical application.

## Background

Gastric cancer is one of the most common gastrointestinal tumors and the second leading cause of cancer-related mortality [1, 2]. As a multifactorial disorders, gastric cancer shows cytological and architectural heterogeneity compared to other gastrointestinal cancers, making it therapeutically challenging. Subpopulation of patients at early-stage of gastric cancers can be cured [3], however most patients diagnose at advanced-stage and show only partial response to standard chemo-agents and targeting-molecules therapy [4, 5]. Therefore, finding new and effective anticancer drugs is of the highest priority.

Schiff bases derived from an amino and carbonyl compound belong to an important class of ligands that coordinated to metal ions. Schiff base-metal compounds have been reported to have promising antibacterial and antitumor activities [6, 7], while the Schiff base coordinated with copper compounds shows the most prominent in the class of molecules [8–13]. Schiff base copper(II) compounds effectively bind to DNA via the DNA grooves, and the binding between the copper ion and DNA is much stronger compared to other essential elements [14]. The potential anticancer effects of Schiff base copper compounds are well reported in various cancer cell lines, including oxidative DNA cleavage to kill cancer stem cell-enriched cells (HMLER-shEcad) and bulk cancer cells (HMLER) [15], inducing apoptosis in lung (A-549) and breast (MDA-MB-231) human cancer cell lines [16], and inducing necrotic cell death and autophagy in breast cancer cells (MCF-7) [17]. The anticancer effects of Schiff base copper compounds are also investigated in in vivo studies. In a diethylnitrosamine-induced liver carcinoma study, Schiff base heterodinuclear copper(II)Mn(II) complex is significantly decreased the incidence and the number of hepatic nodules in a dose-dependent manner via regulating inflammation response and apoptotic pathway [18]. A nephrotoxicity study shows that, whereas cisplatin increases serum urea nitrogen and creatinine levels, no increase in serum biochemical parameters is detected in the animals treated with Schiff base heterodinuclear copper(II)Mn(II) complex [18]. While a major obstacle to widespread use of cisplatin is the persistence of toxic side effects and drug-resistance, Schiff base coordinated copper compounds may be a novel chemotherapeutic strategy for antitumor therapy, with the potential to overcome cisplatin’s shortcoming.

In this study, we synthesized a Schiff base copper coordination compound (SBCCC) and investigated its cell-killing effect and the potential cell death pathway(s) in two gastric cancer cell lines and a xenograft mouse model of gastric cancer.

## Methods

### Cell lines and experimental reagents

Human gastric cancer cell lines, SGC 7901 (TCHu46) and BGC823 (TCHu11), were purchased from the Cell Bank of Shanghai Institute of Biochemistry and Cell Biology, Chinese Academy of Sciences (Shanghai, China). The catalogue number of each cell line is indicated in the bracket following its name. Dulbecco’s modified Eagle’s medium and RPML-1640 culture medium were obtained from Thermo Scientific Co. (USA). The gastric cancer cells, SGC 7901 and BGC823, were maintained in their respective medium in a 5% CO_2_ incubator at 37 °C. 3-(4,5-Dimethylthiazol-2-yl)-5-(3-carboxymethoxyphenyl)-2-(4-sulfophenyl)-2H-tetrazolium (MTS) agent was purchased from Promega Corporation (USA). The annexin V-FITC cell apoptosis detection kit and the cell cycle kit were obtained from Sigma-Aldrich (St. Louis, MO, USA). Acridine orange/ethidium bromide (AO/EB) staining solution, Hoechst 33258, *N*-acetyl cysteine (NAC), and 4,6-diamidino-2-phenylindole, dihydrochloride (DAPI), *N*_ω_-nitro-l-arginine methyl ester (L-NAME), ammonium pyrrolidine dithiocarbamate (PDTC) were purchased from Sigma (St. Louis, MO, USA). Antibodies for detection of nuclear factor (NF)-κBp65, IκB, and p-IκB were obtained from Cell Signaling Technology (Danvers, MA USA). Antibodies for detection of LC3I, LC-3II, and Beclin-1 as well as the secondary antibody were from Shanghai Biological Engineering Company Limited (Shanghai, China).

### Synthesis of the SBCCC

**Fig. 1:**
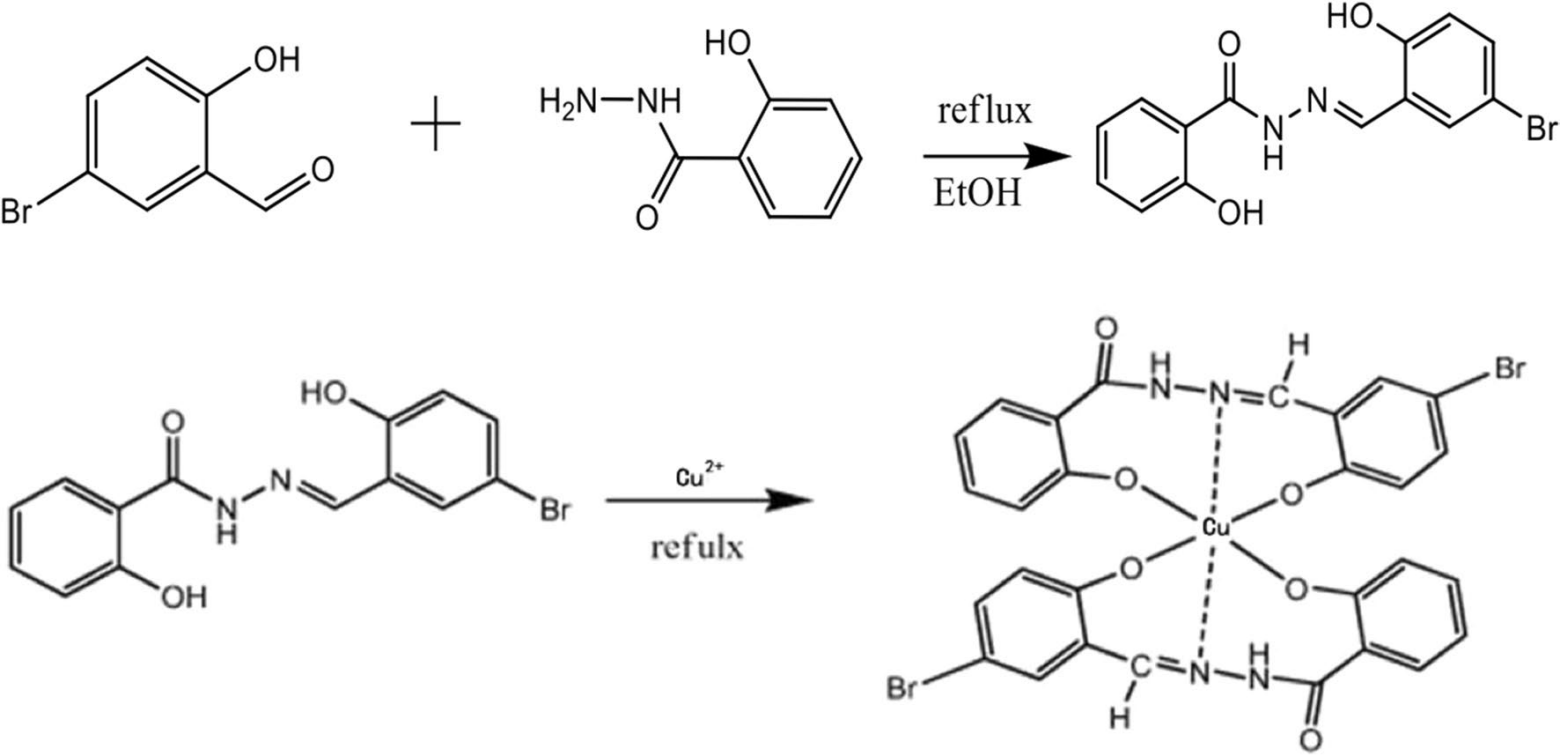
The schematic diagram for synthesis of SBCCC

For synthesis of the SBCCC, 2 mmol of salicylic aldehyde (0.402 g) and 2 mmol of salicylic hydrazide were dissolved in 80 mL of anhydrous ethanol and heated in a water bath under reflux for 7 h. Next, the sediment was filtered at atmospheric pressure and recrystallized twice to afford the Schiff base ligands. The copper coordinated Schiff base complexes were prepared by dissolving the Schiff base ligands (2 mmol) and copper sulfate (1 mmol) in anhydrous ethanol (70 mL). The mixture was heated in a water bath under reflux for 8 h, filtered at atmospheric pressure, and then recrystallized. The synthesis equation for SBCCC was shown in Fig. 1.

### MTS assay

MTS assay was performed to determine cell proliferation and the tested samples were triplicated. In brief, cells were seeded at 1 × 10^4^ cells/well in a 96-well plate. When 80% confluence was reached, the cells were washed with phosphate-buffered saline (PBS) and treated with SBCCC at different concentrations (0.1, 0.2, 0.4, 0.8, 1.6, 3.2, 6.4, and 12.8 μM) diluted in culture medium. After 24 h treatment, 10 μL MTS was added into each well. Optical density (OD) value was determined at 490 nm in each test well using a microplate reader (BioTek, Winooski, VT, USA). Cell growth inhibition rate was calculated according to the equation: cell growth inhibition rate = (1 − OD of SBCCC treated cell/OD of control cell) × 100%. Half maximal inhibitory concentration (IC50) was calculated by GraphPad Prism 7.

### Flow cytometry

Cell death was determined by flow cytometric analysis (FACS Calibur, BD, USA) using an Annexin V-FITC Apoptosis Detection kit according to the manufacturers’ instructions. Cell growth arrest was detected by flow cytometry using propidium iodide (PI) staining. In brief, cells were seeded in a 6-well culture plate at a density of 1 × 10^5^ cells per well. When 80% confluence was reached, the cells were treated with SBCCC at a final concentration of 1 μM. Only culture medium was added as control cells. After treatment for 12, 24, and 36 h, the cells were collected and washed twice with PBS, and incubated with 100 μL of RNase A at 37 °C in a water bath for 30 min. The cells were then incubated with PI at 4 °C in the dark for another 30 min. After completion of all staining steps, the cells were washed with incubation buffer and resuspended in 500ul incubation buffer for flow cytometric analysis. FlowJo 7.6.1 software was used for the data analysis.

### Dual AO/EB, Hoechst 33258 and DAPI staining

For the AO/EB staining and apoptosis analysis, cells were seeded in 6-well culture plates at a density of 1 × 10^4^ cells per well in a 5% CO_2_ incubator at 37 °C. When 80% confluence was reached, the cells were washed with PBS and treated with 200 μL of the SBCCC at 1 μM for 36 h, while only culture medium was added as the control cells. The treated cells were washed with PBS, and then stained with respective solution, 50 μg/mL dual AO/EB solution, 10 μg/mL Hoechst 33258, and 5 μg/mL DAPI were added to each well. The positive staining and cell morphology were examined and photographed using an inverted fluorescence microscope (LX71, Olympus, Japan).

### Monodansylcadaverine (MDC) staining

For the MDC staining, the cells were cultured as described above. The cells were treated with SBCCC at a final concentration of 1 μM for 12, 24 and 36 h. After treatment, the cells were washed with PBS stained with MDC at 50 μM. Cells were incubated for 1 h in a 5% CO_2_ incubator at 37 °C. The positive staining and cell morphology were examined and photographed using an inverted fluorescence microscope (LX71, Olympus, Japan).

### Colony formation assay

For the colony formation assay, SGC-7901 cells and BGC-823 cells were seeded in 6-well culture plates at a density of 1 × 10^4^ cells per well and were cultured in a 5% CO_2_ incubator at 37 °C. When 80% confluence was reached, the cells were washed with PBS and treated with 1 μM SBCCC, 50 μM PDTC, 1 μM SBCCC + 50 μM PDTC respectively for 24 h. After treatment, the cells were collected and reseeded in 6-well plates at a cell density of 200 cells/well. The cells were then cultured for 3 weeks under saturated humidity conditions of 5% CO_2_ at 37 °C, and the culture medium was changed every 3 days. Cell colony formation was examined by hematoxylin staining and the colony numbers were counted.

### Western blot

Total protein was extracted from cells using lysis buffer containing 20 mM Tris–HCl (pH 7.4), 150 mM NaCl, 5 mM EDTA, 1% Triton-X 100, 1% dithiothreitol, and 1% protease inhibitor cocktail. The protein sample was loaded at 40 μg and separated by 10% sodium dodecyl sulfate–polyacrylamide gel electrophoresis and transferred onto a polyvinylidene difluoride membrane (Millipore, USA). Membranes were blocked with 5% (w/v) nonfat dry milk dissolved in Tris-buffered saline plus Tween-20 (TBS-T; 0.1% Tween-20; pH 8.3) at room temperature for 1 h, then incubated with respective primary antibodies (NF-κB p65, 1:150; I κB, 1:1000; p-I κB, 1:1000; LC3I, 1:1000; LC-3II, 1:1000; Beclin-1, 1:1000; Bcl-2, 1:500; Bax, 1:500; caspase-3, 1:1000; cleaved caspase-3, 1:1000; cleaved poly(ADP-ribose) polymerase (PARP), 1:1000; cytochrome C, 1:1000; iNOS, 1:1000; JNK, 1:1000; p-JNK, 1:1000) at 4 °C overnight. After washing with TBS-T, the membranes were incubated with polyclonal anti-horseradish peroxidase-labeled sheep or rabbit IgG (1:5000) secondary antibodies for 2 h at room temperature. Immunobands were visualized using an enhanced chemiluminescence kit (GE Healthcare, USA), according to the manufacturer’s instructions, and exposed to X-ray film. β-Actin was used as a loading control.

### Determination of reactive oxygen species (ROS)

For the measurement of ROS generation, cells were seeded in 6-well culture plates at a density of 1 × 10^4^ cells per well and were cultured in a 5% CO_2_ incubator at 37 °C. When 80% confluence was reached, the cells were washed with PBS and treated with 1 μM SBCCC for 12, 24 and 36 h. For untreated controls, only culture medium were added. After treatment, the cells were washed with PBS and then added 1 mL of freshly prepared DCFH-DA was added at a final concentration of 10 μM. The cells were incubated in the dark for 20 min at 37 °C. The positive staining and cell morphology were examined and photographed using an inverted fluorescence microscope (LX71, Olympus, Japan).

### Xenograft mouse model with gastric cancer cell

Twelve male BALB/c nude mice were purchased from the Guangdong Medical Laboratory Animal Center (Foshan, China). The animals were housed four per cage, given indicated chow and tap water, and maintained at 22 °C and on a 12-h light/dark cycle. To established tumor model, SGC-7901 cells were inoculated subcutaneously at 10^6^ cells/mouse into the right flank. Tumor growth was monitored and tumor sizes were determined by measuring the length and width using a caliber. Tumor sizes were calculated using the formula: tumor volume = length × width × width/2. When the average tumor size reached at 5–6 mm^3^. The 9 tumor-burden BALB/c nude mice were injected with SBCCC at 40 µM/kg bodyweight, i.p., twice per day. Three control mice were treated with same volume saline. The SBCCC treated mice were sacrificed at week 1 and week 2 and the harvested tumor were fixed in formalin. At week 3, the last 3 SBCCC treated mice along with the saline treated controls were sacrificed and the harvested tumor tissues were fixed in formalin for a week, then the tumor size was determined and tumor gross anatomy was photographed. This animal experimental protocol was approved by the Animal Care and Use Committee (College of Basic Medicine, Jilin University).

### Statistical analysis

The experimental data obtained in this study were statistically analyzed using Microsoft Excel 2007 and GraphPad Prism7. The results were statistically analyzed by one-way analysis of variance, and multiple differences were analyzed by Duncan’s test. The results are presented as mean ± standard deviation (SD), and *P* < 0.05 was considered statistically significance.

## Results

### SBCCC inhibits growth of gastric cancer cells

**Fig. 2:**
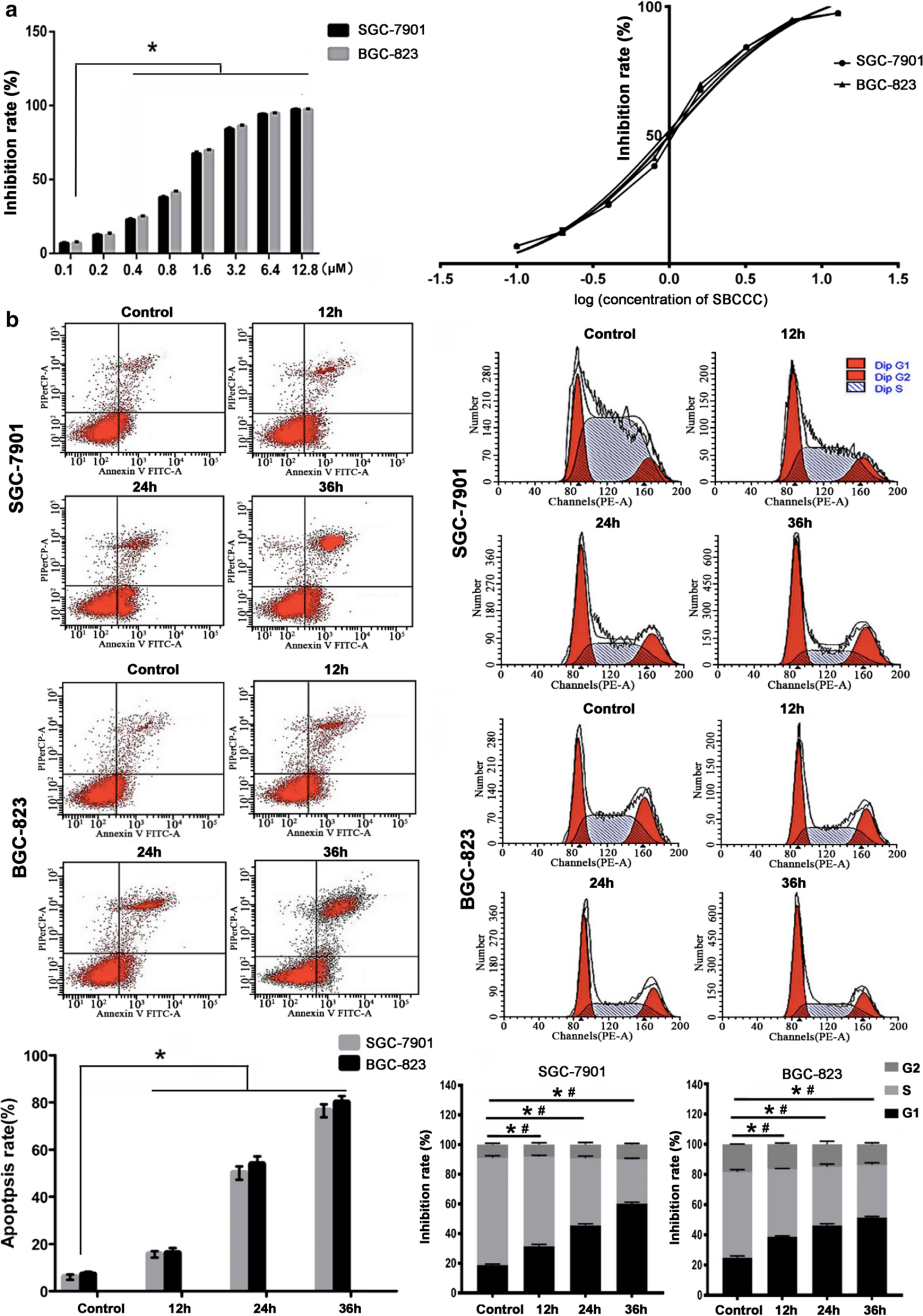
SBCCC-induced cell death and growth arrest in SGC-7901 and BGC-823 cells. **a** Left, MTS cell proliferation assay (*P < 0.05); right, IC50 of SBCCC, calculation based on logarithms of SBCCC concentrations versus the inhibition rates. **b** Left, flow cytometry analysis for apoptosis (*P < 0.05). **b** Right, flow cytometry analysis for cell growth cycle (*P < 0.05 for G1 phase; ^#^*P *< 0.05 for S phase)

With SBCCC treatment, there were significantly increases of inhibitory rates in the gastric cancer cells when SBCCC dosing increased from 0.4 to 12.8 µm (P < 0.05). Both SGC-7901 cells and BGC-823 cells showed a similar growth inhibition trend, in a dose-dependent manner (Fig. 2a, left). IC50 of SGC-7901 cells and IC50 of BGC-823 cells were 1.101 μM and 0.9864 μM, respectively. When the logarithms of SBCCC concentrations were plotted versus the inhibition rates, IC50 showed as 1 μM for both gastric cancer cell lines (Fig. 2a, right). Therefore, we chose 1 μM as an approximate IC50 to continue the further experiments.

### SBCCC induces cell death of gastric cancer cells

To investigate the cellular events, apoptosis and cell growth arrest were determined by Flow cytometry. With SBCCC treatment, the apoptotic rates (%) for SGC-7901 cells were 25.11 ± 1.34 at 12 h, 32.53 ± 2.54 at 24 h, and 53.03 ± 5.67 at 36 h, respectively. Similarly, the apoptotic rates (%) for BGC-823 cells were 28.87 ± 2.34 at 12 h, 39.62 ± 3.61 at 24 h, and 64.33 ± 6.25% at 36 h, respectively. The apoptotic rates for both cell lines significantly increased in a time-dependent manner compared to control cells (*P* < 0.05) (Fig. 2b left). The growth arrest was further evaluated in both SGC-7901 cells and BGC-823 cells with SBCCC treatment. For both cell lines, the percentage of cells in the G1 phase continually increased during the SBCCC treatments for 12, 24, and 36 h. The increases of G1 phase cells were found to be statistically significant compared to control cells (*P* < 0.05). In contrast, the cells in the S phase continually decreased during the SBCCC treatments for 12, 24, and 36 h. The decreases of S phase cells were found to be statistically significant compared to control cells (*P* < 0.05). No changes were observed in the percentage of cells in the G2 phase from both cell lines treated with SBCCC (Fig. 2b right). Based on these results, the growth arrest was happened at G1 phase for both gastric cancer cells, implying that the SBCCC blocked the cell progression to S phase.

### SBCCC interferes with DNA structure in gastric cancer cells

**Fig. 3:**
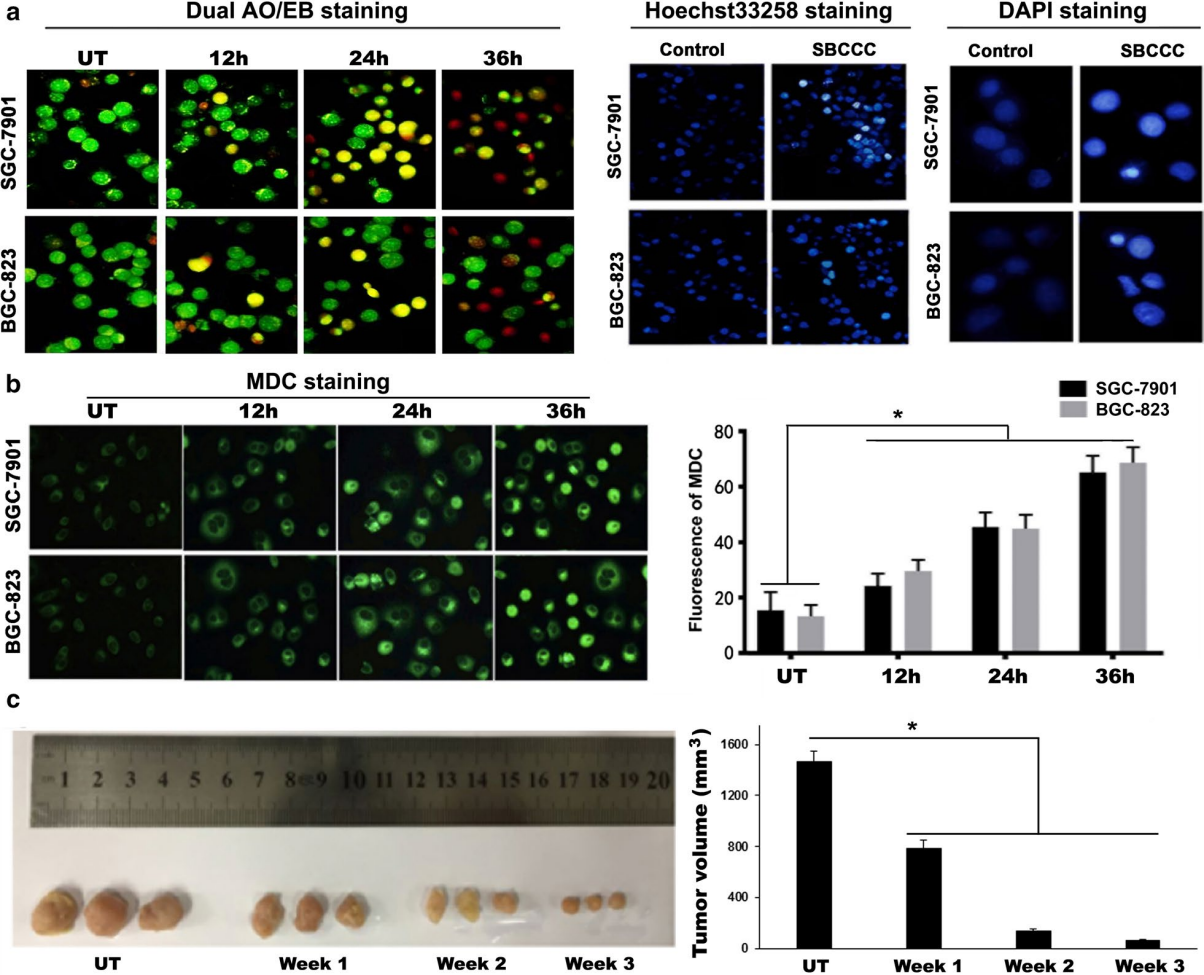
SBCCC-induced apoptosis and autophagy associated cell death in vitro and tumor growth inhibition by SBCCC in vivo. **a** Dual AO/EB staining (×200); Hoechst 33258 (×100) and DAPI staining (×400); **b** MDC staining (×200), h: hour, **c** left, gross anatomy of the tumors harvested from each group of the xenograft mouse model. Right, the tumor volume from control mice and the mice with SBCCC treatment. h: hour; UT: untreated; *P < 0.05

The copper(II) complexes facilitate the DNA cleavage, and exhibited substantial cytotoxic activity against cancer cells [19]. Dual AO/EB, Hoechst 33258 and DAPI staining were performed in both SGC-7901 and BGC-823 cells treated with SBCCC. AO, a cell-permeant dye, emitted green fluorescence when bounded to dsDNA but emitted red fluorescence when bounded to ssDNA. This unique characteristic of AO staining allowed us to evaluate the cell-DNA structure affected by SBCCC. EB only entered cells with damaged membranes and emitting orange-red fluorescence when bound to concentrated DNA fragments. Therefore, dual AO/EB staining allowed us to detect early apoptotic cells, late apoptotic cells, and dead cells. The result indicated that gastric cancer cells with 1 μM SBCCC treatment showed gradually orange color for 12 h. The numbers of orange-stained cells were increased at 24 h, and a portion of the SBCCC treated cells turned to red and orange-red at 36 h (Fig. 3a). Both Hoechst 33358 and DAPI were minor groove-bound and UV-excited. In chromosome condensation or DNA cracking changes, increased binding of Hoechst 33258 dye and DNA shows strong blue compared to normal chromosome cells. DAPI dye is not completely permeability. The ability of permeability for DAPI is improved in apoptotic cells, in which high blue fluorescence is stained in condensed chromosome and cracking DNA, shown abnormal margin of nucleus. When SGC-7901 and BGC-823 cells were treated with 1 μM SBCCC for 36 h, strong blue fluorescence of Hoechst 33258 and DAPI was found. In DAPI staining with high magnification, pyknosis and abnormal margin of nuclei was identified (Fig. 3a). Taken together, SBCCC interfering with DNA structure could be an important mechanism for its cell-killing effect.

### SBCCC promotes autophagy in gastric cancer cells

Monodansylcadaverine (MDC) was reported as a specific in vivo marker for autophagic vacuoles [20]. Therefore, MDC staining was further performed to detect the autophagic vacuoles in both of SGC-7901 and BGC-823 cells challenged by 1 μM SBCCC. The result indicated that the autofluorescent MDC (green fluorescence) gradually increased in the two cell lines, in a time-dependent manner. Computer-image quantification for the MDC green fluorescence indicated that there was significantly increases of the fluorescence intensity of MDC in cancer cells with SBCCC treatment for 12, 24 and 36 h, compared to untreated controls (P < 0.05) (Fig. 3b).

### SBCCC inhibits tumor growth in a xenograft mouse model

Based on the in vitro data, the anti-tumor effect of SBCCC was further tested in vivo using a moue tumor model established by SGC-7901 cells xenograft. The result indicated that SBCCC treated mice showed a significantly decreased in tumor size compared to the untreated mice. As shown in Fig. 3c, the tumor volume in untreated group was 1463 mm^3^ ± 87.79 versus that in the treatment groups 783 mm^3^ ± 66.91 (*P* < 0.05) at week 134 mm^3^ ± 18.82 (*P* < 0.05) at week 2, and 67 mm^3^ ± 6.81 (*P* < 0.05) at week 3.

### Potential signaling targets of cell death by SBCCC

**Fig. 4:**
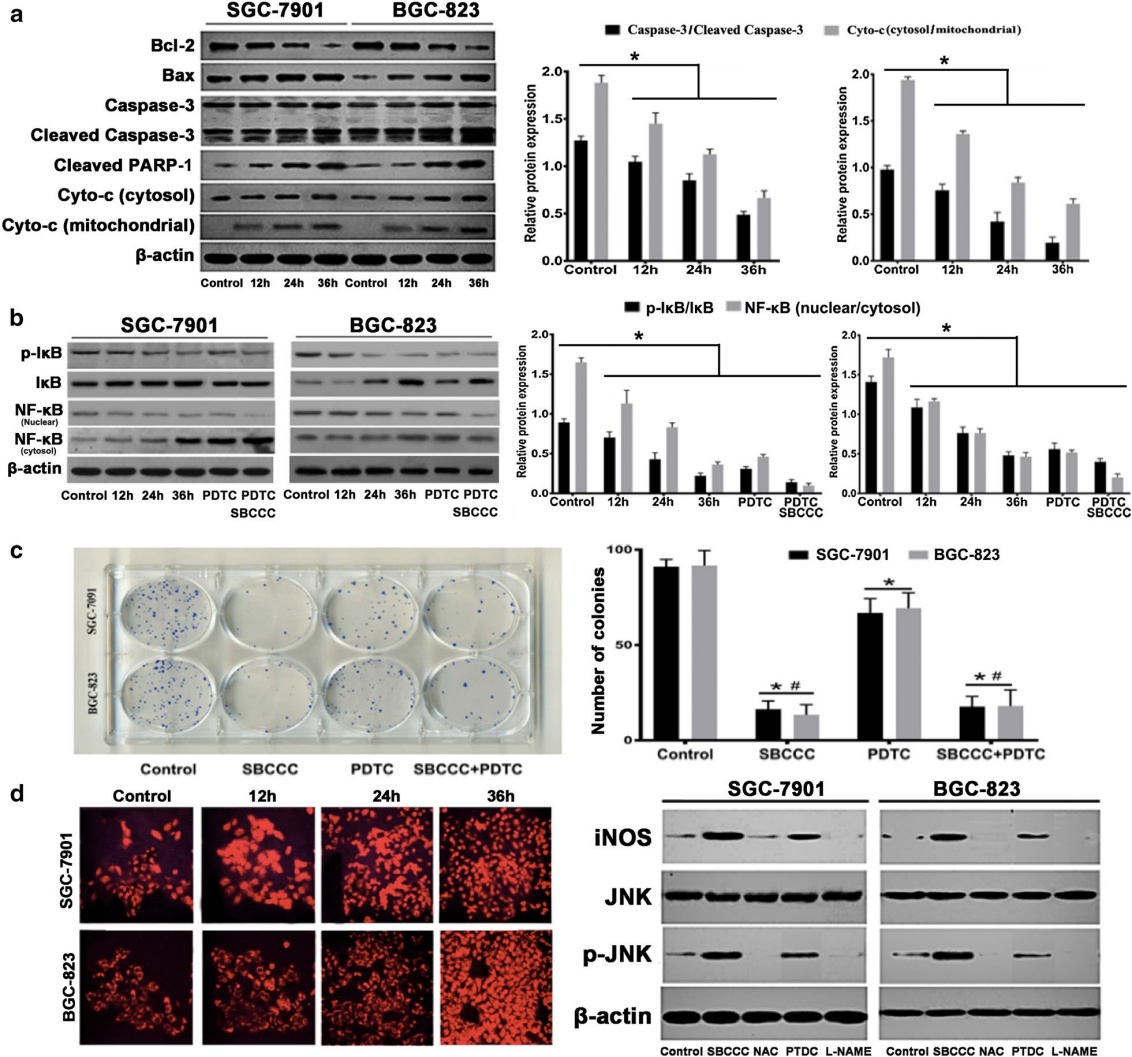
SBCCC induces cell apoptosis via inhibition of NF-κB signaling and ROS production. **a** Western blot analysis for protein levels of Bcl-2, Bax, caspase-3, cleaved caspase-3, cleaved PARP-1, and cytochrome C (Cyto-c). h: hour; *P < 0.05. **b** Western blot analysis for protein levels of p-IκB, IκB and NF-κB. h: hour; *P < 0.05. **c** Cell colony formation assay. h: hour; *P < 0.05 vs control; ^#^*P *< 0.05 vs PDTC. **d** Left, ROS production detected by DCFH-DA staining. Right, Western blot analysis for protein levels of iNOS, JNK, and p-JNK. h: hour

As the SBCCC induced apoptosis had been demonstrated by above experiments, we further investigated the important components of apoptotic signaling in term of apoptotic initiators and effectors to elucidate the potential anti-tumor mechanism(s). As expected, SBCCC treatment significantly reduced the protein level of Bcl-2 but increased the protein levels of Bax, and reduced the release of cytochrome C from mitochondria to cytosolc, in a time-dependent manner in both cell lines. Moreover, leaved caspase-3, and cleaved PARP-1, a hallmark of apoptosis [21], were found significantly increased in the SBCCC treated gastric cancer cells compared to the controls (Fig. 4a). Because activation of NF-κB signaling was well established in gastric cancer [22], the critical components in NF-κB signaling were further evaluated in the two gastric cancer cell lines challenged by SBCCC. Because pyrrolidine dithiocarbamate (PDTC) could prevent degradation of I-κB and translocation of NF-κB from the cytoplasm into the nucleus [23], it was used a selective NF-κB inhibitor to investigate if the anti-cancer effect of SBCCC was mediated through NF-κB inhibition. The results indicate that treatments with either 1 μM SBCCC or 50 µM PDTC significantly inhibited (*P* < 0.05) I-κB phosphorylation and NF-κB nucleus translocation (Fig. 4b), indicating that the SBCCC caused cell death, at least in part, was mediated by inhibition of NF-κB signaling because of less anti-apoptosis products being produced. However, the cell colony formation number in the cells with PDTC treatment was not as less as that in the cells with SBCCC treatment (Fig. 4c), indicating that other pathways might contribute to the cell death by SBCCC, in addition to its NF-κB inhibitory effect. Previous report showed that the copper(II) complexes induced apoptosis could be dependent on ROS generation [24]. Therefore, we further examined the generation of ROS in the gastric cancer cells with SBCCC treatment. As presented in Fig. 4d, ROS generation in SGC-7901 and BCG-823 cells was significantly increased in a time-dependent manner. SBCCC treatment significantly increased the expressions of iNOS and phosphorylated JNK and the increased expressions were inhibited by two inhibitors, *N*-acetyl-l-cysteine (NAC, an inhibitor of ROS) and L-NAME (an inhibitor of NOS) but not the NF-κB inhibitor PDTC.

**Fig. 5:**
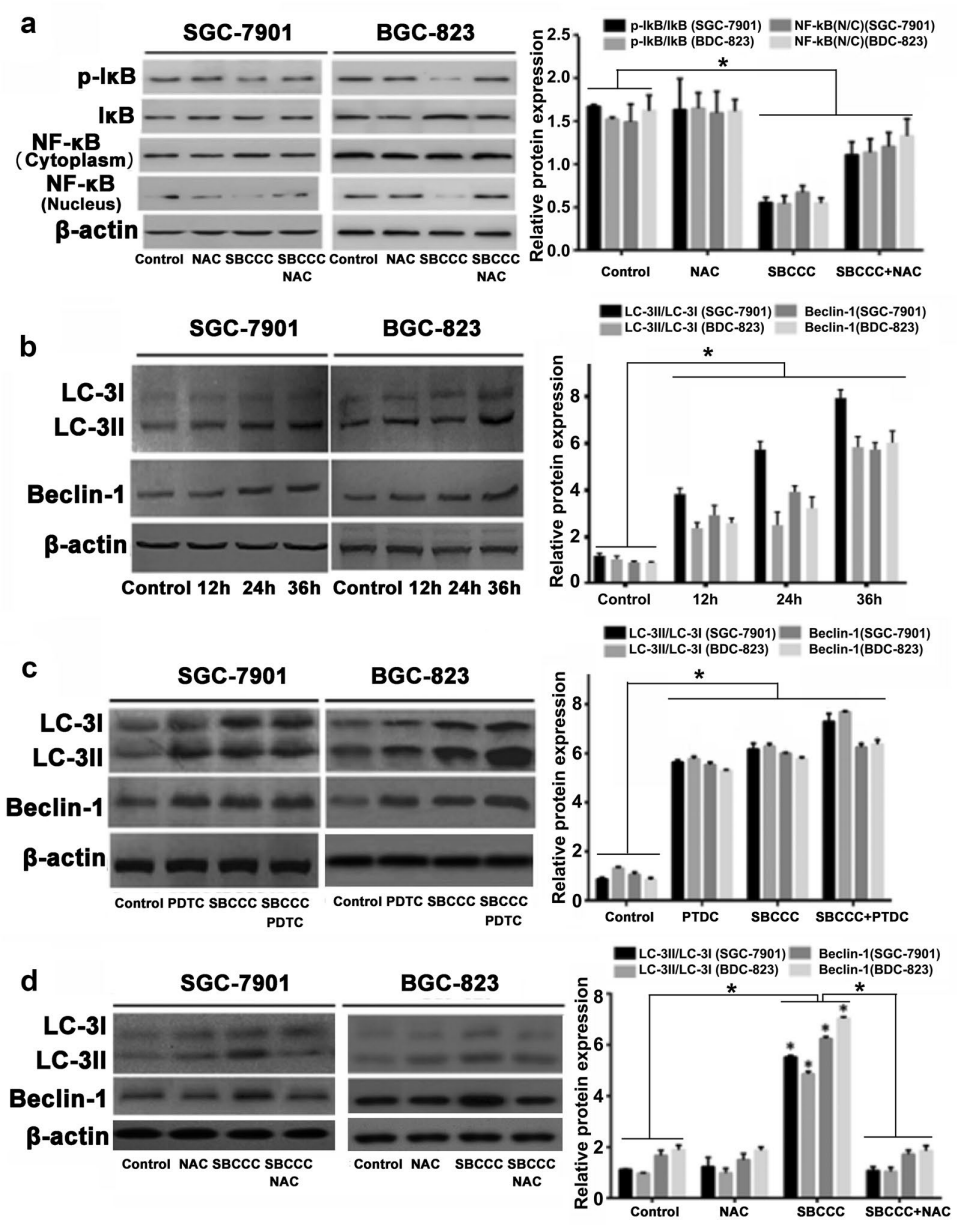
SBCCC induced autophagy in ROS-dependent signaling. **a** Western blot analysis for protein levels of p-IκB, IκB and NF-κB in SGC-7901 and BCG-823 cells treated with SBCCC, NAC and combination of SBCCC and NAC. *P < 0.05. **b** Western blot analysis for protein levels of LC3-I, LC-3II, and Beclin-1 in SGC-7901 and BCG-823 cells treated with SBCCC for 12 h, 24 h and 36 h. h: hour; *P < 0.05. **c** Western blot analysis for protein levels of LC3-I, LC-3II, and Beclin-1 in SGC-7901 and BCG-823 cells treated with SBCCC, PDTC and combination of SBCCC and PDTC. *P < 0.05. **d** Western blot analysis for protein levels of LC3-I, LC-3II, and Beclin-1 in SGC-7901 and BCG-823 cells treated with SBCCC, NAC and combination of SBCCC and NAC. *P < 0.05. *N* Nucleus; *C* Cytoplasm

NF-κB was reported as a pleiotropic transcription factor activated by low levels of ROS but inhibited by antioxidants [25]. Further experiment was performed to determine if the SBCCC induced apoptosis could be ROS-dependent in addition to NF-κB signaling. Interestingly, the ROS inhibitor (NAC) alone or in combination with SBCCC did not significantly affect NF-κB signaling (Fig. 5a). Although ROS could act as upstream signaling of the NF-κB pathway, our results indicated that the cell-killing effect of SBCCC were from both ROS-dependent DNA cleavage and inhibition of NF-κB signaling. As that SBCCC induced increases of autophagic vacuoles (MDC staining), the important molecular targets of autophagy were further evaluated. The protein expressions of Beclin-1 and LC-3II were significantly increased in SGC-7901 and BGC-823 cells with SBCCC treatment compared to the control cells (Fig. 5b). To further elucidate the autophagy signal linking to NF-κB or ROS pathways, NAC and PDTC were used in combination with the SBCCC to treat SGC-7901 and BGC-823 cells. PDTC did not affect SBCCC induced increases of Beclin-1 and LC-3II (Fig. 5c), but NAC significantly attenuated the SBCCC induced protein expressions of Beclin-1 and LC-3II (Fig. 5d). These results suggested that SBCCC associated autophagy was ROS-dependent but not via the NF-κB signaling.

## Discussion

Metal-based compounds were widely used in the treatment of diseases [26]. The discovery of cisplatin in 1960 was a milestone in the history of metal-based compounds used in the treatment of cancers [27]. Cisplatin is highly reactive and covalently binds to DNA to form DNA-cisplatin adducts, which in turn induce DNA damage, inhibit cell replication, and induce apoptosis. Unfortunately, the side-effects such as peripheral neuropathy and nephropathy were concerned for clinical use, and the tumor cells could develop resistance mechanisms to those platinum drugs by repairing the DNA damage [28]. In past decade, there was a shift in the research focus of metal-based antitumor drugs towards copper [29], an essential element of the human body showing less toxicity during tumor treatment [30]. Copper coordinated compounds were different from platinum in many aspects, including physiological distribution in the body, intracellular aggregation properties, inhibiting cell proliferation, rendering the potential of copper-based with less toxicity and avoiding resistance [31]. In this study, a new synthesized SBCCC was investigated in regards of its antitumor properties in two gastric cancer cell lines as well as a xenograft mouse model of gastric cancer. The IC50 of SBCCC for the two gastric cancer cells was 1 µM, which was much less than cisplatin IC50 which ranged from 2.5 to 50 µM in multiple human cancer cell lines [32]. The lower IC50 could render SBCCC with the potential of less side-effect for clinical application. The SBCCC induced cell death was demonstrated by multiple techniques, including flow cytometry, dual AO/EB staining, Hoechst 33258 staining, and DAPI staining. The SBCCC-treated cells showed cytoplasmic shrinkage, membrane blebbing, and DNA fragmentation, which were the signature features of apoptosis reported by previous studies [33, 34].

Similar to the NF-κB inhibitor PDTC, SBCCC treatment significantly inhibited the NF-κB transactivation for the productions of apoptotic initiator and effector, including Bcl-2, Bcl-xL, cleaved caspase-3, and cleaved PARP-1. Our results agreed with previous reports in which inhibition of NF-κB transactivation by Schiff base-derived metal complex [35, 36]. It was reported that ROS induced apoptosis via destruction of mitochondrial membranes, release of cytochrome C from mitochondria, the downstream activation of the caspase system ensues [37–39]. In our study, ROS was produced in the gastric cancer cells with SBCCC treatment, indicating that ROS production was a significant mechanism contributing to cell death. Using inhibitors to block both ROS and NF-κB, we demonstrated that the cell-killing effect of SBCCC was attributed to not only mitochondrial apoptosis directly triggered by ROS-dependent DNA cleavage, but also amplified apoptotic signaling via inhibition of NF-κB transactivation. ROS-dependent autophagy was reported as a novel strategy to kill multidrug-resistant cancer cells [40, 41]. A previous study showed that copper(II) complex induced cell death via ROS-mediated autophagy [42]. The ROS-dependent autophagy by SBCCC was demonstrated in this study. When treated with ROS inhibitor NAC, the SBCCC induced activation of autophagy was totally abolished. ROS acted as upstream signaling for the autophagy pathway activation with SBCCC treatment to induce the autophagy associated cell death. The potential anti-tumor mechanisms of SBCCC were addressed in this study including (1) inhibition of NF-κB signaling; (2) ROS production; and (3) autophagy. Our data suggested that SBCCC could be a novel agent to overcome the multidrug-resistance of cancer cells, through various cell-killing mechanisms.

However, other mechanisms of SBCCC could also contribute to cell death in addition to inhibition of NF-κB, ROS production and autophagy. For example, a study proposed that the net effect of Schiff bases copper(II) complex for cell death could be destruction of the structural integrity of cell membranes, regardless of apoptosis [43]. Schiff base copper complexes with ternary structure could also serve as ligand to inhibit proteasome and to induce apoptosis [44]. Nevertheless, further studies are needed to investigate SBCCC in regards of other mechanisms and potential clinical application.

## Conclusion

The synthesized SBCCC induced cancer cell death via inhibition of NF-κB, ROS production and autophagy. The multiple cell-killing mechanisms were important to overcome therapeutic failure because of multidrug-resistance of cancer cells. SBCCC, with a lower IC50 compared to cisplatin, could render it the potential to overcome the side-effect for clinical application.

## Abbreviations

SBCCC: Schiff base copper coordinated compound
AO/EB: acridine orange/ethidium bromide
NAC: *N*-acetyl cysteine
L-NAME: *N*(ω)-nitro-l-arginine methyl ester
DAPI: 4,6-diamidino-2-phenylindole, dihydrochloride
PDTC: pyrrolidine dithiocarbamate
MDC: monodansylcadaverine
PARP: poly(ADP-ribose) polymerase
ROS: reactive oxygen species

## References

[1] Ferlay J, Shin HR, Bray F, Forman D, Mathers C, Parkin DM (2010). Estimates of worldwide burden of cancer in 2008: GLOBOCAN 2008. Int J Cancer.

[2] Siegel RL, Miller KD, Jemal A (2017). Cancer statistics, 2017. CA Cancer J Clin.

[3] Kim GH (2016). Endoscopic submucosal dissection for early gastric cancers with uncommon histology. Clin Endosc.

[4] Strong VE, DAmico TA, Kleinberg L, Ajani J (2013). Impact of the 7th edition AJCC staging classification on the NCCN clinical practice guidelines in oncology for gastric and esophageal cancers. J Natl Compr Canc Netw.

[5] Hayes T, Smyth E, Riddell A, Allum W (2017). Staging in esophageal and gastric cancers. Hematol Oncol Clin North Am.

[6] Wang PH, Keck JG, Lien EJ, Lai MM (1990). Design, synthesis, testing, and quantitative structure-activity relationship analysis of substituted salicylaldehyde Schiff bases of 1-amino-3-hydroxyguanidine tosylate as new antiviral agents against coronavirus. J Med Chem.

[7] Cai T, Xian M, Wang PG (2002). Electrochemical and peroxidase oxidation study of N’-hydroxyguanidine derivatives as NO donors. Bioorg Med Chem Lett.

[8] Hajrezaie M, Golbabapour S, Hassandarvish P, Gwaram NS, MohdAli H, Majid N, Abdulla MA (2012). Acute toxicity and gastroprotection studies of a new schiff base derived copper(II) complex against ethanol-induced acute gastric lesions in rats. PLoS ONE.

[9] Pontiki E, Hadjipavlou-Litina D, Chaviara AT (2008). Evaluation of anti-inflammatory and antioxidant activities of copper(II) Schiff mono-base and copper(II) Schiff base coordination compounds of dien with heterocyclic aldehydes and 2-amino-5-methyl-thiazole. J Enzyme Inhib Med Chem.

[10] Chaviara AT, Cox PJ, Repana KH, Papi RM, Papazisis KT, Zambouli D, Kortsaris AH, Kyriakidis DA, Bolos CA (2004). Copper(II) Schiff base coordination compounds of dien with heterocyclic aldehydes and 2-amino-5-methyl-thiazole: synthesis, characterization, antiproliferative and antibacterial studies. Crystal structure of CudienOOCl2. J Inorg Biochem.

[11] Chaviara AT, Cox PJ, Repana KH, Pantazaki AA, Papazisis KT, Kortsaris AH, Kyriakidis DA, Nikolov GS, Bolos CA (2005). The unexpected formation of biologically active Cu(II) Schiff mono-base complexes with 2-thiophene-carboxaldehyde and dipropylenetriamine: crystal and molecular structure of CudptaSCl2. J Inorg Biochem.

[12] Bolos CA, Nikolov GS, Ekateriniadou L, Kortsaris A, Kyriakidis DA (1998). Structure-activity relationships for some diamine, triamine and schiff base derivatives and their copper(II) complexes. Met Based Drugs.

[13] Sorenson JR (1989). Copper complexes offer a physiological approach to treatment of chronic diseases. Prog Med Chem.

[14] Burkitt MJ (1994). Copper–DNA adducts. Methods Enzymol.

[15] Lu C, Eskandari A, Cressey PB, Suntharalingam K (2017). Cancer stem cell and bulk cancer cell active copper(II) complexes with vanillin Schiff base derivatives and naproxen. Chemistry.

[16] Paul A, Hazra S, Sharma G, Guedes da Silva MFC, Koch B, Pombeiro AJL (2017). Unfolding biological properties of a versatile dicopper(II) precursor and its two mononuclear copper(II) derivatives. J Inorg Biochem.

[17] Konarikova K, Perdikaris GA, Gbelcova H, Andrezalova L, Sveda M, Ruml T, Laubertova L, Reznakova S, Zitnanova I (2016). Autophagy in MCF-7 cancer cells induced by copper complexes. Pharmacol Rep.

[18] Demirci S, Dogan A, Basak N, Telci D, Dede B, Orhan C, Tuzcu M, Sahin K, Sahin N, Ozercan IH (2015). A Schiff base derivative for effective treatment of diethylnitrosamine-induced liver cancer in vivo. Anticancer Drugs.

[19] Chityala VK, Sathish Kumar K, Macha R, Tigulla P (2014). DNA cleavage, cytotoxic activities, and antimicrobial studies of ternary copper(II) complexes of isoxazole Schiff base and heterocyclic compounds. Bioinorg Chem Appl.

[20] Biederbick A, Kern HF, Elsasser HP (1995). Monodansylcadaverine (MDC) is a specific in vivo marker for autophagic vacuoles. Eur J Cell Biol.

[21] Chaitanya GV, Steven AJ, Babu PP (2010). PARP-1 cleavage fragments: signatures of cell-death proteases in neurodegeneration. Cell Commun Signal.

[22] Sokolova O, Naumann M (2017). NF-kappaB signaling in gastric cancer. Toxins (Basel).

[23] Cuzzocrea S, Chatterjee PK, Mazzon E, Dugo L, Serraino I, Britti D, Mazzullo G, Caputi AP, Thiemermann C (2002). Pyrrolidine dithiocarbamate attenuates the development of acute and chronic inflammation. Br J Pharmacol.

[24] Zhou XQ, Li Y, Zhang DY, Nie Y, Li ZJ, Gu W, Liu X, Tian JL, Yan SP (2016). Copper complexes based on chiral Schiff-base ligands: DNA/BSA binding ability, DNA cleavage activity, cytotoxicity and mechanism of apoptosis. Eur J Med Chem.

[25] Curtin JF, Donovan M, Cotter TG (2002). Regulation and measurement of oxidative stress in apoptosis. J Immunol Methods.

[26] Amolegbe SA, Akinremi CA, Adewuyi S, Lawal A, Bamigboye MO, Obaleye JA (2017). Some nontoxic metal-based drugs for selected prevalent tropical pathogenic diseases. J Biol Inorg Chem.

[27] Jungwirth U, Kowol CR, Keppler BK, Hartinger CG, Berger W, Heffeter P (2011). Anticancer activity of metal complexes: involvement of redox processes. Antioxid Redox Signal.

[28] Martin LP, Hamilton TC, Schilder RJ (2008). Platinum resistance: the role of DNA repair pathways. Clin Cancer Res.

[29] Gandin V, Porchia M, Tisato F, Zanella A, Severin E, Dolmella A, Marzano C (2013). Novel mixed-ligand copper(I) complexes: role of diimine ligands on cytotoxicity and genotoxicity. J Med Chem.

[30] Tardito S, Bassanetti I, Bignardi C, Elviri L, Tegoni M, Mucchino C, Bussolati O, Franchi-Gazzola R, Marchio L (2011). Copper binding agents acting as copper ionophores lead to caspase inhibition and paraptotic cell death in human cancer cells. J Am Chem Soc.

[31] Tisato F, Marzano C, Porchia M, Pellei M, Santini C (2010). Copper in diseases and treatments, and copper-based anticancer strategies. Med Res Rev.

[32] Song H, Li W, Qi R, Yan L, Jing X, Zheng M, Xiao H (2015). Delivering a photosensitive transplatin prodrug to overcome cisplatin drug resistance. Chem Commun (Camb).

[33] Hu W, Kavanagh JJ (2003). Anticancer therapy targeting the apoptotic pathway. Lancet Oncol.

[34] Ly JD, Grubb DR, Lawen A (2003). The mitochondrial membrane potential (deltapsi(m)) in apoptosis; an update. Apoptosis.

[35] Hajrezaie M, Paydar M, Moghadamtousi SZ, Hassandarvish P, Gwaram NS, Zahedifard M, Rouhollahi E, Karimian H, Looi CY, Ali HM (2014). A Schiff base-derived copper (II) complex is a potent inducer of apoptosis in colon cancer cells by activating the intrinsic pathway. Sci World J.

[36] Shawish HB, Wong WY, Wong YL, Loh SW, Looi CY, Hassandarvish P, Phan AY, Wong WF, Wang H, Paterson IC (2014). Nickel(II) complex of polyhydroxybenzaldehyde N4-thiosemicarbazone exhibits anti-inflammatory activity by inhibiting NF-kappaB transactivation. PLoS ONE.

[37] Thakor P, Subramanian RB, Thakkar SS, Ray A, Thakkar VR (2017). Phytol induces ROS mediated apoptosis by induction of caspase 9 and 3 through activation of TRAIL, FAS and TNF receptors and inhibits tumor progression factor Glucose 6 phosphate dehydrogenase in lung carcinoma cell line (A549). Biomed Pharmacother.

[38] Bishayee K, Ghosh S, Mukherjee A, Sadhukhan R, Mondal J, Khuda-Bukhsh AR (2013). Quercetin induces cytochrome-c release and ROS accumulation to promote apoptosis and arrest the cell cycle in G2/M, in cervical carcinoma: signal cascade and drug-DNA interaction. Cell Prolif.

[39] Zhu Y, Jiang Y, Shi L, Du L, Xu X, Wang E, Sun Y, Guo X, Zou B, Wang H (2017). 7-O-Geranylquercetin induces apoptosis in gastric cancer cells via ROS-MAPK mediated mitochondrial signaling pathway activation. Biomed Pharmacother.

[40] Xu Z, Jiang H, Zhu Y, Wang H, Jiang J, Chen L, Xu W, Hu T, Cho CH (2017). Cryptotanshinone induces ROS-dependent autophagy in multidrug-resistant colon cancer cells. Chem Biol Interact.

[41] Dewaele M, Maes H, Agostinis P (2010). ROS-mediated mechanisms of autophagy stimulation and their relevance in cancer therapy. Autophagy.

[42] Guo WJ, Ye SS, Cao N, Huang J, Gao J, Chen QY (2010). ROS-mediated autophagy was involved in cancer cell death induced by novel copper(II) complex. Exp Toxicol Pathol.

[43] Choi EJ, Lee JI, Kim GH (2012). Evaluation of the anticancer activities of thioflavanone and thioflavone in human breast cancer cell lines. Int J Mol Med.

[44] Zhang Z, Bi C, Schmitt SM, Fan Y, Dong L, Zuo J, Dou QP (2012). 1,10-Phenanthroline promotes copper complexes into tumor cells and induces apoptosis by inhibiting the proteasome activity. J Biol Inorg Chem.

